# Therapeutic Methods

**Published:** 1889-03

**Authors:** 


THERAPEUTIC METHODS. By Jabez P. Dake, M. D. Pages 195. Otis Clapp & Son, Boston and Providence.

This is a second edition of Dr. Dake's little volume. It gives first a brief sketch of medical history from the time of Pythagoras and Hippocrates down to Hahnemann. Next follows a *résumé* of the requisites for a medical education. In part second we find *therapeutics* proper considered. Part third is devoted to the "Demands of Similia." These last are considered from Dr. Dake's peculiar standpoint. His well-known views upon drug proving and upon posology and pharmacy are given.

